# Precautionary behaviours of individuals with multimorbidity during the COVID-19 pandemic

**DOI:** 10.1007/s10433-021-00632-8

**Published:** 2022-01-04

**Authors:** Alice Delerue Matos, Andreia Fonseca de Paiva, Cláudia Cunha, Gina Voss

**Affiliations:** 1 Department of Sociology, Institute of Social Sciences, University of Minho, Braga, Portugal; 2 Communication and Society Research Centre, Institute of Social Sciences, University of Minho, Braga, Portugal

**Keywords:** COVID-19, SHARE, Precautionary Behaviours, Multimorbidity, Pandemic, Public Health

## Abstract

Studies show that older individuals with multimorbidity are more susceptible to develop a more severe case of COVID-19 when infected by the virus. These individuals are more likely to be admitted to Intensive Care Units and to die from COVID-19-related conditions than younger individuals or those without multimorbidity. This research aimed to assess whether there are differences in terms of precautionary behaviours between individuals aged 50 + with multimorbidity and their counterparts without multimorbidity residing in 25 European countries plus Israel. We used data from the SHARE-COVID19 questionnaire on the socio-demographic and economic characteristics, multimorbidity, and precautionary behaviours of individuals. SHARE wave 8 and 7 databases were also used to fully identify individuals with multimorbidity. Our results showed that individuals with multimorbidity were more likely to exhibit precautionary behaviours than their counterparts without multimorbidity when gender, age, education, financial distress and countries were included as controls. Additionally, we found that women, more educated individuals and those experiencing more financial distress adopt more protective behaviours than their counterparts. Our results also indicate that the prevalence of precautionary behaviours is higher in Spain and Italy and lower in Denmark, Finland and Sweden. To guarantee the adoption of preventive actions against COVID-19, public health messaging and actions must continue to be disseminated among middle and older aged persons with multimorbidity, and more awareness campaigns should be targeted at men and less educated individuals but also at persons experiencing less financial distress, particularly in countries where people engaged in fewer precautionary behaviours.

## Introduction

Emerging infectious diseases that progress rapidly from a local epidemic to a pandemic are a significant threat to global public health. The most recent example, the COVID-19 pandemic, has impelled the authorities to intervene quickly and effectively to reduce the dissemination of the virus. A critical element to limit the spread of the disease is rapid and large-scale behavioural changes. And, indeed, authorities worldwide have responded with a set of guidelines aimed at changing people’s behaviours. These guidelines include vigorous hand washing, physical distancing, staying at home as much as possible, maintaining respiratory hygiene, wearing a face mask, avoiding touching the face with unclean hands and disinfecting frequently touched objects and surfaces (Ejaz et al., 2020; Shankar et al., 2020). Although these guidelines are aimed at everyone, they should be especially followed by individuals who are at greater risk of suffering complications from the disease when infected by the virus.

A growing body of evidence suggests that the risk of developing severe life-threatening complications in COVID-19 pandemic is higher, namely in individuals who are older and/or suffering from multimorbidity (Ramage-Morin & Polsky, 2020; Sanyaolu et al., 2020; Zaninotto et al., 2020), defined by Marengoni (2011) as the co-existence of two or more chronic conditions. When infected, these individuals have an increased likelihood of hospitalization, admission to intensive care units (ICU) and even death (Boddington et al., 2020; Docherty et al., 2020; Froes et al., 2020; Guan et al., 2020; Iaccarino et al., 2020; Zhang et al., 2020).

The theoretical background on behavioural research in response to pandemics is heavily focused on theories of risk perception, such as the Health Belief Model (HBM) (Strecher et al., 1997). According to the HBM, whether or not an individual will choose to act on a health-related behaviour will build on the perception of four aspects: susceptibility to a health threat; severity of said threat; likelihood of reducing the threat by engaging in health-related behaviour, and costs or barriers that can be associated with engaging in said behaviour. HBM predicts that higher perceived threat leads to a higher likelihood of engagement in health-promoting behaviours (Glanz & Bishop, 2010; Strecher et al., 1997). In a study by Jose et al. (2021) using the HBM to characterize the perceptions and behavioural change of individuals regarding COVID-19 and control measures, the authors found that individuals who practised hand washing, measures that prevent the transmission of infection, perceive that contracting COVID-19 would be more serious and more susceptible. They also found that older adults who had comorbid conditions also reported that contracting COVID-19 would be very severe.

The concept of Self-efficacy, defined by Bandura (1997) as the conviction that one can successfully execute the behaviour required to produce the outcomes, was later added to the HBM as a separate concept (Rosenstock et al., 1988) and it is now considered an important element to help initiate and maintain a conduct. According to the HBM that includes the self-efficacy concept (Champion & Skinner, 2002), individuals need to perceive susceptibility and severity to a said threat, believe that the change and/or behaviour will bring benefits and must also perceive themselves as capable to overcome barriers.

There are other theories of risk perception, such as the Protection Motivation Theory (PMT) (Rogers & Prentice-Dunn, 1997) that can provide useful structures to understand behavioural choices during global challenges. In a review of literature, Bish & Michie (2010) found studies that point out that factors of the PMT, such as perceived severity of a threat, perceived vulnerability to said threat and response-efficacy, were linked to protective behaviours against infectious diseases.

A common underlying idea of these theories is that, in the presence of a threat, the individuals who perceive themselves to be more vulnerable to said threat, will increasingly engage in risk-prevention behaviours (Bonem et al., 2015; Wise et al., 2020). Similarly, individuals who perceive themselves as having a lower risk of developing illness are more likely to engage in unhealthy, risky behaviours (Jose et al., 2021). Understanding theories of behaviour changes is important to implement interventions that aim at promoting healthy behaviours and improving effective public health programs (Glanz & Bishop, 2010).

Therefore, health problems, including multimorbidity, may lead to a perception of increased vulnerability to the serious repercussions of the COVID-19 disease, consequently motivating greater precautionary behaviours to avoid infection (Jose et al., 2021).

In addition to the health condition, research outcomes elucidate the relationship between individual demographic and socioeconomic characteristics, on the one hand, and precautionary behaviours during pandemics, on the other hand.

Several researchers explain that women are slightly more afraid of the infection and perceive the disease as more severe, implementing more protective behavioural changes (Cvetković et al., 2020; Kim & Crimmins, 2020; Lep et al., 2020; Lüdecke & von dem Knesebeck, 2020; Zickfeld et al., 2020). Moreover, older men are less worried about the COVID-19 pandemic and were less cautious, according to a study conducted by Barber et al. (2020).

In a literature review on determinants of precautionary behaviours during pandemics, Bish and Michie (2010) conclude that the relationship between age and precautionary behaviours is not completely clear, although most studies point that increasing age is associated with a greater likelihood of adopting precautionary behaviours.

The level of education and the income, which point to the social position of the individual, are some of the characteristics that are often taken into account in the analysis of the social determinants of health behaviours. But, regarding education, the pattern of findings in pandemic studies is not straightforward. While some studies indicate that having a higher educational level is correlated with more preventive behaviours during pandemics (Lüdecke & von dem Knesebeck, 2020; Zickfeld et al., 2020), others reveal the opposite results (Tang & Wong, 2005) or no association at all (Tang & Wong, 2003).

Income is considered in a much smaller number of studies on health behaviours in the context of a pandemic. In research conducted in the United States of America (USA), Jay et al. (2020) concluded that residents of low-income neighbourhoods were less likely to stay at home, even with state orders, due to the need to work outside the home.

We aim at describing whether the adoption of precautionary behaviours differs among individuals aged 50 + with and without multimorbidity, in 25 European countries and Israel. Inspired by the risk perception theories, the underlying hypothesis is that individuals with multimorbidity behaved more cautiously than those without multimorbidity, controlling for main demographic and socio-economic characteristics of the individuals that are associated with precautionary behaviours. Since precautionary behaviours also depend on the characteristics of the context in which individuals operate, the variability of countries is also taken into account.

This study goal is to fill a gap in knowledge about precautionary behaviours during a pandemic. Studies comparing two population groups with different health risks, such as this one involving individuals with and without multimorbidity, on the health behaviours adopted in a pandemic context are scarce. Furthermore, there is a lack of studies on Europe and comparative studies between countries. This research is, as far as we know, the first European cross-national study of precautionary behaviours during the first wave of the COVID-19 pandemic, involving a very significant number of countries.

## Data and methods

### Study design and setting

The current study uses data from the SHARE-COVID19 (release 0) mainly, but also from wave 8 (release 0) and 7 (release 7.1.0) databases, that were used to identify individuals with multimorbidity. For more methodological details, please see Börsch-Supan (2020a, b), Börsch-Supan et al. (2013), Bergmann et al. (2019) and Scherpenzeel et al. (2020). The sample was restricted to respondents aged 50 + who answered “yes” to the routing question “Since the outbreak of Corona, have you ever left your home?”, as those were the only ones who answered all the precautionary behaviours questions. Individuals who never left home were therefore excluded from this study. They represent 18.5% of the SHARE-COVID19 respondents. Hence, the sample size for this study is 41534 individuals.

This study focuses on 25 European countries plus Israel. The Netherlands was excluded due to the lack of observations in our interest variable.

### Measures

#### Outcome variable 

The precautionary behaviours considered in this research were: (1) going shopping; (2) going out for a walk; (3) meeting more than 5 people from outside the household; (4) visiting other family members since the outbreak (answers were reclassified into two groups: About the same/More often, and Not anymore/Less often). Additional precautionary behaviours were (5) wearing a face mask in public; (6) keeping distance from others in public (answers were reclassified into two groups: Always/Often, and Sometimes/Never). Finally, some more precautionary behaviours were assessed with the yes/no questions: (7) washed hands more than usual; (8) used hand sanitizer or disinfectant fluids more than usual; and (9) covered coughs and sneezes more than usual. A person was categorized as having high precautionary behaviours if she/he reported 7 or more of the above-mentioned behaviours and having low precautionary behaviours if fewer than 7 behaviours were reported. The cut point was based on the median value of the precautionary behaviours, by country.

#### Interest variable

Multimorbidity was defined as reporting two or more chronic conditions (Marengoni et al., 2011). This study uses the following chronic conditions: hip fracture; diabetes or high blood sugar; high blood pressure or hypertension; heart attack including myocardial infarction or coronary thrombosis or any other heart problem including congestive heart failure; chronic lung disease such as chronic bronchitis or emphysema; cancer or malignant tumour, including leukaemia or lymphoma, but excluding minor skin cancers; another illness or health condition. These conditions were reported in the SHARE-COVID19 questionnaire, using the questions “Since we last interviewed you, were you diagnosed with a major illness or health condition?” and “Do you have any of the following illnesses or health conditions?”. Since individuals may suffer from non-recent multimorbidity, if they answered “no” to the first question above, the same questions from wave 8 or wave 7 were used to fully classify respondents into two groups: with multimorbidity and without multimorbidity.

#### Control variables

The control variables in the model were selected based on the literature review. The gender and age of the respondent at the time of the interview were selected as control variables. To take into account the socioeconomic position of the respondents, two indicators were used: education and financial distress. Education was measured according to the highest level of education attained using the standardized coding of the International Standard Classification of Education (ISCED-97). This variable was categorized into three groups: low education (ISCED-97 levels 0–2 corresponding to lower secondary school at the most); medium education (ISCED-97 level 3, upper secondary school) and high education (ISCED-97 levels 4–6 corresponding to post-secondary school). Since income was not available in the SHARE-COVID19 database and it is an indicator that can vary substantially in a short period of time, therefore discouraging the use of information reported in previous waves, we used a proxy indicator of income, available in SHARE-COVID19: financial distress, assessed by the question “Thinking of your household's total monthly income since the outbreak of Corona, would you say that your household is able to make ends meet?”. The answers were reclassified into two groups: “with great difficulty”/ “with some difficulty”, and “fairly easily”/ “easily”.

### Statistical analyses

This study was carried out in two stages. Firstly, to characterize our study population, descriptive statistics were applied using calibrated individual weights, since the SHARE survey does not have a uniform sample design. To analyse whether there are differences between the high and low precautionary behaviour groups, statistical tests for two-group comparison were performed (t test (t) and chi-square tests (X^2^)). To complement these analyses, we used Cohen’s d/Phi effect size measure to assess the magnitude of the observed effect on our sample. Confidence Intervals (CI) for these observed effect measures were also calculated.

Secondly, to examine the association between multimorbidity and precautionary behaviours, a multilevel logistic regression, with individuals as level one and countries as level two, was performed. As a first step, the null model (Model 1) was tested as a means to determine the variance of precautionary behaviours that are explained by country differences. The Intraclass Correlation Coefficient (ICC) of the null model is 13.6 per cent, higher than the recommended cut-point of 5 per cent and, for this reason, we used multilevel modelling (LeBreton & Senter, 2008). As a second step, the model was adjusted for the confounders (age, gender, education, and financial distress) (Model 2), with continuous variables centred. We did not control for mental health in our statistical models, as the multimorbidity variable already includes affective problems and chronic neurodegenerative diseases, such as Alzheimer’s disease. As the last step, we added our interest variable, multimorbidity (Model 3). The deviance statistic is used to test if additional model predictors do improve the fit of the model. Odds ratios (OR), 95% confidence intervals (IC) and significance (where p values of < 0.05 were considered statistically significant) are presented in the tables below. Statistical analyses were conducted using R software, version 4.0.2, and IBM SPSS Statistics 25.

## Results

In our sample, the mean age of the participants was 65.99 years (SD = 9.43) and women constituted 52.60 per cent of the sample. In addition, 31.30 per cent completed primary education or less, 40.61 per cent completed secondary education and 28.09 per cent completed post-secondary education. Moreover, across the study sample, 30.69 per cent of the respondents reported being financially distressed. Altogether 40.97 per cent of the participants reported the presence of multimorbidity, and 71.82 per cent of the respondents indicated having high precautionary behaviours.

Characteristics of the low and high precautionary behaviours groups are displayed in Table 1. Among the respondents who reported multimorbidity, 72.2% engaged in high precautionary behaviours. Without controlling for confounders, all the variables listed in Table 1, except age, differed statistically in the two groups of individuals with high and low precautionary behaviours, although with no significant effect size.

**Table 1:** Characteristics of low and high precautionary behaviours groups

	low precautionary behaviours	high precautionary behaviours	Cohen´s *d*			
	(*N* = 12,930)	(*N* = 28,602)	*t*/χ^2^	P value	/ phi	(CI 95%)
Age, mean(SD)	66.47 (9.75)	65.89 (9.29)	0.947	0.344	0.01	–0.0110.031
*Gender*						
Male (%)	53.75	44.90	388.219	< 0.001	0.10	0.0870.106
Female (%)	46.26	55.10				
*Education*						
Primary or less (%)	24.9	33.83	151.751	< 0.001	0.06	0.0510.070
Secondary (%)	46.16	38.41				
Post-secondary (%)	28.94	27.76				
*Financial distress*						
No (%)	74.56	32.73	280.767	< 0.001	0.08	0.0720.092
Yes (%)	25.44	58.59				
*Multimorbidity*						
No (%)	60.14	58.59	116.211	< 0.001	0.05	0.0430.062
Yes (%)	39.86	41.41				

Source: Preliminary SHARE wave 8, release 0. Conclusions are preliminary. Weighted data, N = 41,534

Notes: t/χ^2^ (t-test and chi-squared test), CI (confidence intervals). Tests for effect size: Cohen’s d: small effect (≥ 0.20); medium effect (≥ 0.50); large effect (≥ 0.80); Phi: small effect (≥ 0.10); medium effect (≥ 0.30); large effect (≥ 0.50). Significant associations (p < 0.05) are in bold. The sample was limited to individuals aged 50 + who have left home since the outbreak of COVID-19

**Fig. 1:**
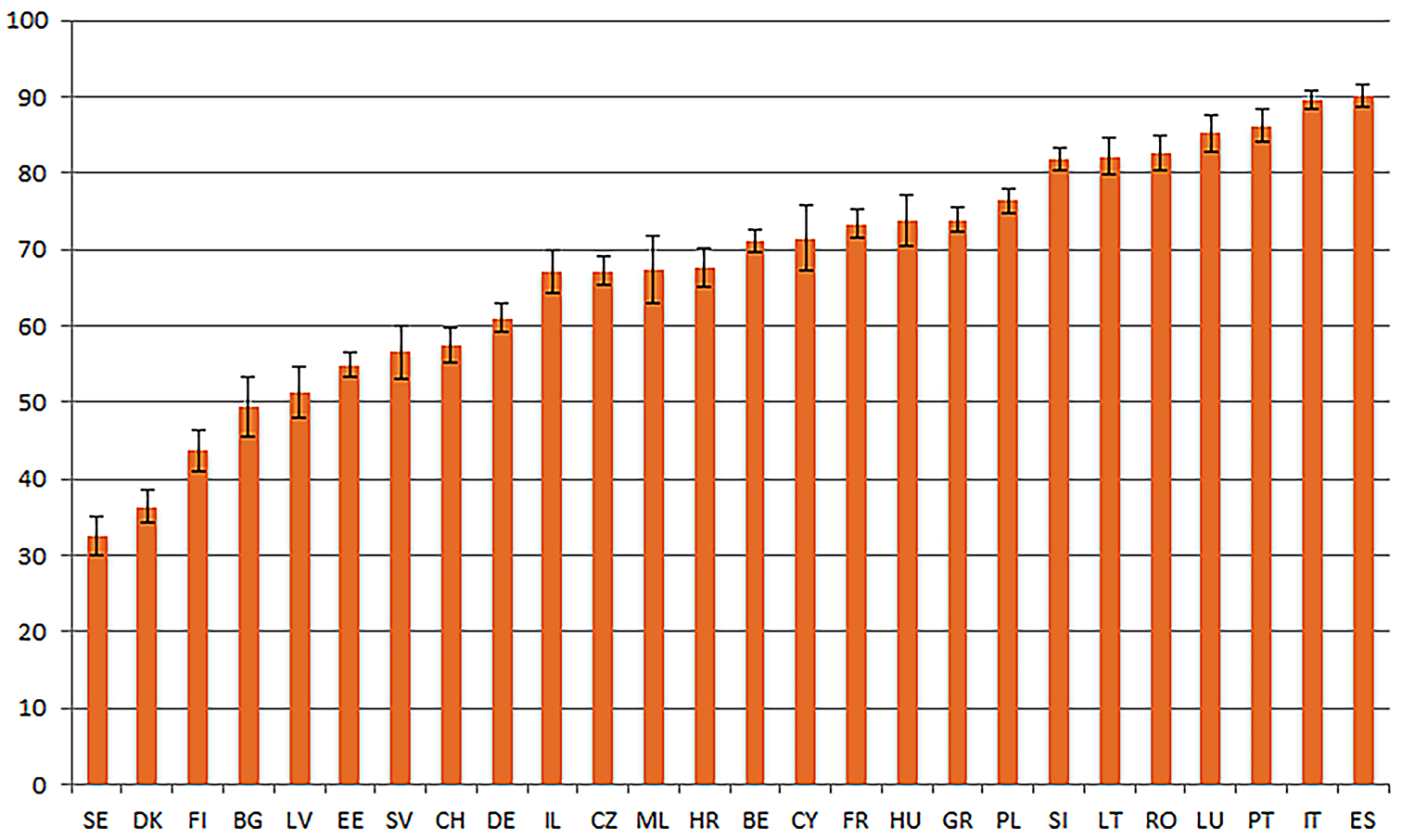
Prevalence of high precautionary behaviours by country Source: Preliminary SHARE, wave 8, release 0. Conclusions are preliminary. Notes: *SE* Sweden; *DK* Denmark; *FI* Finland; *BG* Bulgaria; *LV* Latvia; *EE* Estonia; *SV* Slovakia; *CH* Switzerland; *DE* Germany; *IL* Israel; *CZ* Czech Republic; *ML* Malta; *HR* Croatia; *BE* Belgium; *CY* Cyprus; *FR* France; *HU* Hungary; *GR* Greece; *PL* Poland; *SI* Slovenia; *LT* Lithuania; *RO* Romania; *LU* Luxembourg; *PT* Portugal; *IT* Italia; *ES* Spain. Brackets denote 95% confidence intervals. The sample was limited to individuals aged 50 + who have left home since the outbreak of COVID-19

Figure 1 shows the prevalence of high precautionary behaviours by country. Overall, the highest prevalence of precautionary behaviours was reported in Spain and Italy (90.02 and 89.49 per cent, respectively), while the lowest was reported in Sweden, Denmark, and Finland (32.59; 36.36 and 43.64 per cent, respectively).

The results of the multilevel logistic regression for precautionary behaviours are shown in Table 2. When Model 2 was compared with the null model, the deviance lowered, which means that, by adding the control variables, the model increased its quality (44,221.4, *p* value < 0.001). When multimorbidity was included (Model 3), decreases in deviance were also observed (42,717.2, *p* value < 0.001).

**Table 2:** Multilevel logistic regressions for precautionary behaviours

	Model 1	Model 2	Model 3
	OR (CI 95%)	OR (CI 95%)	OR (CI 95%)
*Fixed parts*			
(Intercept)	2.41 (1.80–3.21) ***	1.61 (1.21–2.15) **	1.45 (1.08–1.94)*
Age (years)		1.03 (1.01–1.06) **	1.01 (0.98–1.03)
Female		1.63 (1.56–1.71) ***	1.63 (1.55–1.70) ***
*Education*			
Primary or less		ref.	ref.
Secondary		1.07 (1.01–1.14) *	1.08 (1.01–1.15) *
Post-secondary		1.21 (1.13–1.29) ***	1.24 (1.16–1.32) ***
Financial distress		1.20 (1.13–1.27) ***	1.11 (1.11–1.25) ***
Multimorbidity			1.25 (1.20–1.32) ***
*Random parts*			
ICCcountry	0.136	–	–
Deviance	47,809.9	44,221.4	42,717.2
N countries	26	26	26

Source: Preliminary SHARE, wave 8, release 0. Conclusions are preliminary

*Ref* reference group, *OR* odds ratio, *CI* confidence intervals, *ICC* Intra-class Correlation Coefficients. Significant associations: ´***´ < 0,001; '**' < 0,01; '*' < 0,05. The sample was limited to individuals aged 50 + who have left home since the outbreak of COVID-19

The final model (Model 3) showed that individuals with multimorbidity presented 25 per cent higher chances of having high precautionary behaviours compared with respondents without multimorbidity (OR = 1.25, 95% CI [1.20;1.32]).

Women had 63 per cent more chances to engage in high precautionary behaviours compared to men (OR = 1.63, 95% CI [1.55; 1.70]). Respondents with secondary and post-secondary education had more chances of adopting high precautionary behaviours, compared to respondents with primary education (OR = 1.08, 95% CI [1.01; 1.15]; OR = 1.24, 95% CI [1.16; 1.32], respectively). This is also the case for respondents who reported financial distress (OR = 1.11, 95% CI [1.11; 1.25]) compared to their counterparts.

## Discussion

Our results showed that individuals with multimorbidity were more likely to engage in precautionary behaviours than their counterparts. This result is consistent with the latest report from the English Longitudinal Study of Ageing (ELSA) COVID-19 Substudy, where individuals with multimorbidity had more precautionary behaviours, more specifically being isolated and staying at home (Zaninotto et al., 2020). In another recent study by Laires et al (2020), it was found that older Portuguese individuals with and without underlying health conditions had more self-awareness for the severity of COVID-19 and adopted more precautionary behaviours. Our results might be explained by theories of risk perception. Previous research into people’s behaviours during pandemics concluded that the adoption of protective behaviours is consistent with these theories that point that people who perceive themselves as being more vulnerable to a threat tend to protect themselves more and engage in more precautionary behaviours (Bish & Michie, 2010; Rogers & Prentice-Dunn, 1997; Strecher et al., 1997; Wise et al., 2020). Therefore, self-awareness and perception of risk might explain why people with multimorbidity engage in more protective behaviours. Nevertheless, our conclusions differ from studies developed in the USA and Canada (O’Conor et al., 2020; Ramage-Morin & Polsky, 2020; Wolf et al., 2020), which showed that individuals with underlying health conditions were not engaging in more precautionary behaviours than their counterparts. However, it should be borne in mind that these studies were conducted at an early stage of the pandemic, comprise small sample sizes and cover specific geographical areas or use samples that are not representative of the older population. Another possible explanation for both of these results, meaning whether people engage or not in protective behaviours, might also be related to the underlying concept of self-efficacy (Bandura, 1997) integrated into a more recent version of the Health Belief Model. As stated before, for behaviour to occur individuals need to believe that the change and/or behaviour will bring benefits but they also must perceive themselves as capable of overcoming barriers, thus having self-efficacy.

Concerning gender, our results are in line with previous studies from authors who found that women adopted more protective behaviours against COVID-19, compared to men (Cvetković et al., 2020; Kim & Crimmins, 2020; Lep et al., 2020; Lüdecke & von dem Knesebeck, 2020). The existing literature on risk perception can explain these results, as it states that women and men differ in their perception of risk (Gustafson, 1998).

Following the theories of risk perception, even though it would be expected that older adults would engage in more precautionary behaviours (Barber & Kim, 2020; Laires et al., 2020), our results showed no age differences between individuals with high and low precautionary behaviours.

Regarding education, our results are in line with studies that indicate that having a higher educational level is correlated with more precautionary behaviours (Bish & Michie, 2010; Zickfeld et al., 2020). Another interesting result is that the respondents who reported being more financially distressed had more chances of adopting more precautionary behaviours than those who did not indicate that they were financially distressed. We hypothesise that for individuals who are more financially distressed, it might be more difficult to afford medical assistance and medication, and therefore they adopted more measures to avoid becoming infected. This is, nevertheless, a research question to be investigated in the future.

In our descriptive analysis by country (Fig. 1), we found that in Sweden, Denmark and Finland, the prevalence of precautionary behaviours was low. On the other hand, the prevalence was higher in Spain and Italy. Our hypothesis for these results lies in the prevalence of COVID-19 infection and mortality in Spain and Italy during the first wave of the COVID-19 pandemic. Since these countries had a worse experience of the pandemic, their residents were more likely to engage in precautionary behaviours because they may have perceived themselves as being more likely to get infected with COVID-19. Furthermore, the multiple health guidelines and restrictive measures imposed by the government may have been more effective in giving individuals perception of the severity of the risk. In contrast, during the first wave of the COVID-19 pandemic, Sweden, Denmark and Finland were not as severely affected, and so the guidelines and restrictive measures imposed were not as stringent, which gave individuals a weaker sense of risk perception. Another possible explanation for these country differences might be attributed to citizen’s trust in their governments to handle the pandemic. According to the Eurobarometer 2020, in Sweden, Denmark and Finland's citizens report higher satisfaction with government measures, while in Spain and Italy the satisfaction is lower (Standard Eurobarometer 93: Summer 2020—Data Europa EU). Thus, this trust of Swedish, Danish and Finish citizens in their governments may have restricted their actions against the virus to the public health guidelines while Italian and Spanish citizens may have felt the need to take additional measures to protect themselves against the pandemic.

Since the beginning of the pandemic, COVID-19 has caused increased morbidity and mortality around the globe, putting individuals with multimorbidity at a higher risk of significant harm. This study shows that public health messaging and actions must continue to be disseminated among middle and older aged individuals with multimorbidity to guarantee that this population continues to take preventive actions against COVID-19. Additionally, more awareness campaigns should be aimed at middle and older aged men and individuals with less education and people with less financial distress, since these groups adopt fewer precautionary behaviours. The same is true for countries whose population adopts fewer precautionary behaviours, to make individuals aware of the risk, protect themselves and others and help mitigate the impact of the virus, regardless of their risk category.

Finally, yet importantly, with COVID-19, fast and large-scale behavioural changes are urgent and make it crucial for policymakers to be aware of whether the recommended guidelines are being followed or if there is a necessity for more awareness campaigns targeting people at greater risk of developing a more severe case of the disease. Thus, the results of this study are critical for obtaining a clear understanding of people’s adoption of protective behaviours during the pandemic. This is essential for communication strategies and for addressing the present and future health crisis.

## Strength and limitations

To our knowledge, this is the first study analysing the association between multimorbidity and engaging in precautionary behaviours in middle and older-aged Europeans in a cross-national perspective. Our sample is representative of the European and Israeli population, which allows us to perform generalizations. However, the findings of this research need to be interpreted within a framework sensitive to the limitations of the study. Since this study uses a cross-sectional design, and the temporality of association is a strong criterion for causality, we cannot assume the presence of causality, but rather, help generate a causal hypothesis. Furthermore, considering that obesity is now recognized as one of the main risk factors for COVID-19 severity, another limitation is the fact that we had no possibility of including this health condition in our study as the information was not collected. Finally, we were not able to consider the individuals who never left home as we do not have information that allows us to distinguish between those who stayed at home as an act of very high protection against the virus and those who did it because of health problems (e.g. bedridden).

## Conclusion

This study has several potential implications for middle and older aged individuals with multimorbidity, indicating that public health messages and guidelines should continue and should be reinforced to target this group as they are at a higher risk of developing worse health outcomes due to COVID-19. Since more pandemics may arise in the future, it is important to identify and target specific vulnerable groups in the early stages of an outbreak in order to help contain and mitigate the spread, and also avoid increased morbidity and mortality. Public health safety messages should continue to be disseminated among the population, particularly in some countries.

## Data Availability

See share-project.org.
